# Assessment of serum biomarker changes following the COVID-19 pandemic and vaccination: a cohort study in Sylhet, Bangladesh

**DOI:** 10.3389/fpubh.2025.1435930

**Published:** 2025-02-21

**Authors:** Shangida Akther, Fairoz Samiha, Sabrina Amita Sony, Mohammad Anamul Haque, Mohammad Abul Hasnat, S. M. Saiful Islam, Shamim Ahmed, Mohammad Abdullah-Al-Shoeb

**Affiliations:** ^1^Department of Biochemistry and Molecular Biology, School of Life Sciences, Shahjalal University of Science and Technology, Sylhet, Bangladesh; ^2^Department of Statistics, School of Physical Sciences, Shahjalal University of Science and Technology, Sylhet, Bangladesh; ^3^Department of Chemistry, School of Physical Sciences, Shahjalal University of Science and Technology, Sylhet, Bangladesh

**Keywords:** SARS-CoV-2 infection, COVID-19 vaccine, hyperglycemia, lipid profile, serum creatinine

## Abstract

**Objectives:**

Coronavirus 2019 (COVID-19) has spread throughout the world and the current COVID-19 vaccines have shown to be the most effective means of combating the COVID-19. This study focused to examine the status of serum biomarkers in individuals infected and non-infected with SARS-CoV-2, both before and after COVID-19 pandemic and vaccination.

**Methods:**

This study comprised 133 adults aged 35 and older including both academic and non-academic personnel associated with Shahjalal University of Science and Technology in Sylhet, Bangladesh. Participants were evaluated before and after COVID-19 pandemic, as well as following two doses of vaccination. Blood samples were collected to measure different serum biomarkers, including fasting blood sugar (FBS), serum creatinine, serum alanine transaminase (ALT), total cholesterol (TC), triglyceride (TG), Low density lipoprotein-cholesterol (LDL-C), and High density lipoprotein-cholesterol (HDL-C). Statistical analysis was performed using SPSS software.

**Result:**

In all participants, serum creatinine, FBS and TC levels significantly increased after two doses of vaccination (*p* = 0.022, 0.006, 0.05) compared to pre-vaccination levels. Notably, all serum biomarkers showed a significant elevation (*p* ≤ 0.05) in the self-reported SARS-CoV-2 infected group (*n* = 44). Additionally, 31% of participants were newly diagnosed with hyperglycemia after receiving the COVID-19 vaccine.

**Conclusion:**

The findings indicate that both self-reported SARS-CoV-2 infection and COVID-19 vaccination could influence different serum biomarker levels. However, further comprehensive research is necessary to discern the precise factors contributing to the alterations observed in the serum biomarker levels for future health management strategy.

## Introduction

The world has been recently ravaged by COVID-19, caused by the novel severe acute respiratory syndrome coronavirus 2 (SARS-CoV-2) virus. Designated a pandemic by the world health organization (WHO) on March 11, 2020, COVID-19 swiftly spread to over 100 countries, resulting in 6.5 million deaths by October 2022 (1, 2). Coronaviruses lead to various disorders, including acute respiratory stress syndrome (ARDS), respiratory failure, and systemic consequences. This marks the third coronavirus outbreak in the late twentieth century, following Severe acute respiratory syndrome coronavirus-1 (SARS-CoV-1) and Middle East respiratory syndrome coronavirus (MERS-CoV), potentially sharing harmful mechanisms (3, 4).

COVID-19’s primary features include widespread lung damage and sudden respiratory failure (5), while effects on other organs require investigation. The virus can enter the bloodstream from lung infection, affecting the kidneys and causing renal cell destruction. COVID-19 RNA is detected in 15% of patients’ plasma via reverse transcription polymerase chain reaction (RT-PCR) (5). 6.7% of SARS patients experienced acute kidney injury (AKI), with a 91.7% death rate among AKI patients (6). Over 40% of SARS-CoV-2-infected individuals display renal issues, including elevated serum creatinine and blood urea nitrogen (BUN) levels (7). SARS outbreak in 2003 and the COVID-19 pandemic affected various organs, including the gastrointestinal tract, liver, and kidneys (8). SARS-CoV-2 binds to diverse liver receptors like angiotensin-converting enzyme 2 (ACE2), found in hepatocytes and cholangiocytes. The Transmembrane protease serine 2 (TMPRSS2) is expressed in multiple cell types, and endoprotease Furin is expressed universally. These receptors suggest direct viral-induced liver damage in COVID-19 (9). Severe cases exhibit higher liver enzyme levels (10) and liver damage varies from 14 to 53% in COVID-19 patients (11), likely due to SARS-CoV-2’s affinity for ACE2 (12). Multiple organ failure (MOF) seems to be another potential causative factor of liver injury in COVID-19 patients (13, 14).

Multiple retrospective studies reveal varying mortality rates among COVID-19 patients (15, 16). Older patients with conditions like diabetes, hypertension, or coronary heart disease face worse outcomes (17). Recent research shows a link between blood sugar and COVID-19 (18, 19). Hyperglycemia in COVID-19 patients leads to a 7.3% mortality rate, significantly higher than the 0.9% in those with normal glucose levels (20). About 14 to 32% of COVID-19-infected diabetes mellitus patients are at high risk to develop severe to critical illnesses (21).

One of the essential approaches for limiting the COVID-19 pandemic has been rapid and widespread SARS-CoV-2 immunization. Recent mRNA vaccines, such as BNT162b2 (Pfizer- BioNTech) and mRNA-1273 (Moderna-NIAID), have given adequate defense against severe COVID-19 infection (22, 23). However, some immune-mediated responses, such as glomerulonephritis and autoimmune hepatitis, have been reported following vaccination (24, 25). The first COVID-19 vaccine had administered in Bangladesh on January 27, 2021, and the country’s major immunization campaign began on February 7, 2021 (26).

A biomarker serves as a detectable indication of disease severity or presence and is used to mark certain health conditions (27). They play a crucial role in diagnosing and predicting major illnesses such as diabetes and heart disease. Combining multiple biomarkers can offer a comprehensive health profile, aiding in early detection and disease prevention (28).

Noncommunicable diseases (NCDs), also referred to as chronic diseases, that cannot be transmitted directly from one person to another, are typically long-lasting and arise from a combination of genetic, physiological, environmental, and behavioral factors (29, 30). The incidence of various NCDs such as diabetes, renal disease, liver dysfunction, and cardiovascular disease are more prevalent among adults 30 years of age and older (31, 32), and the WHO reports that most NCD deaths occur before the age of 70 (33). Faculty members and employees from Shahjalal University of Science and Technology (SUST), Sylhet with age 35 or above constituting the study’s participants may or may not be immune to the risks associated with these diseases; indeed, some may already be afflicted with these ailments and may have also contracted COVID-19. All study participants have received vaccination. Therefore, biomarker tests can offer valuable insights into the overall health status of SUST faculty members.

This study aimed to analyze biomarker levels following COVID-19 pandemic and vaccination, comparing these levels with pre-infection and pre-vaccination data. Additionally, it compared the data between infected and non-infected participants. Fasting blood sugar was measured for hyperglycemia prognosis, serum creatinine for kidney function, lipid profile for hyperlipidemia prevalence, and Alanine transaminase for liver function using collected serum samples before and during the COVID-19 pandemic in three different phases. The study’s insights could inform about COVID-19 pandemic and vaccination effects and offer some recommendations for future health management and disease prevention.

## Methods

### Data source, study area, and participants

This population-based research study was carried out at the Department of Biochemistry and Molecular Biology, Shahjalal University of Science and Technology (SUST) in Sylhet, Bangladesh. The study utilized data collected as part of a large-scale cohort study conducted from November 2019 to April 2022, which aimed to assess the overall health status of academic and non-academic staff of SUST, as well as the impact of the SARS-CoV-2 pandemic and vaccination. We invited 35 years and older staff of SUST to participate in this research study, requiring 8–10 h of fasting. In the first phase, 154 staff participated in this study from November 2019 to February 2020, before the COVID-19 pandemic. We commenced the second round of sample collection with the same group of participants, from February 2021 to April 2021. During this phase, 181 participants accepted our invitation and we collected their samples with a modified questionnaire to assess the impact of COVID-19 on them. The third and final phase of sample collection took place from October 2021 to April 2022. In this phase, 391 participants joined in this study. Notably, at the time of sample collection, all the infected participants had recovered from COVID-19 and had received two doses of the vaccine. Data from 133 participants were included in this study based on specific inclusion criteria: (1) Data collected from participants before the COVID-19 pandemic and vaccination, (2) Individuals aged 35 and above, (3) Individuals with chronic diseases but controlled with medicine, and (4) Fasting condition. The exclusion criteria: (1) Pregnant women, (2) Chain smoker, (3) Having immediate serious side effects after vaccination. If any participant could not adequately answer all questions or express their physical status in clear terms, their data was excluded. The hemolyzed blood sample was also discarded, and additional analysis was carried out to see if there was any uncertainty over the biomarker level. The study was approved by the Ethical Review Committee of the Biochemistry and Molecular Biology Department at SUST, under approval code 02/BMB/2018. Written informed consent was obtained from all participants before the study began, and all procedures followed relevant guidelines and regulations.

### Data collection

A standard questionnaire was developed in both English and Bengali (the native language) based on a thorough literature review. The questionnaire included questions on anthropometric measurements, socio-demographic information, food habits, self-report of SARS-CoV-2 infection, and vaccination details. Research students and technicians received training from the research supervisor and team leader on interviewing study participants and the data collection process. Subsequently, they gathered participants’ anthropometric and socio-demographic data through interviews. Participants’ weight and height were measured following standard procedures detailed elsewhere (34, 35). In brief, body weight was recorded to the nearest 0.1 kg using modern electronic digital LCD weighing scales (Beurer 700, Germany). Height was measured to the nearest 0.1 cm using a height-measuring tape while participants stood erect. Body mass index (BMI) was calculated as weight in kilograms divided by height in meters squared (kg/m^2^). The questionnaire also addressed participants’ SARS-CoV-2 infection status, including whether they were clinically diagnosed via RT-PCR test of nasal swab, as well as their health precautions during the COVID-19 pandemic.

Health precautions were categorized into two groups. The “strictly maintained” category includes consistently and properly wearing masks in crowded spaces and on public transport, maintaining a minimum distance of 6 feet, regularly using hand sanitizer and soap, exercising frequently, and avoiding large gatherings. In contrast, “roughly maintained” precautions involve wearing masks sporadically, attending gatherings occasionally, and practicing hygiene less frequently. Additionally, participants were asked about their COVID-19 vaccination status, specifically whether they had received a single dose, two doses, or remained unvaccinated.

### Blood sample collection and biochemical analysis

A 5 mL blood sample was drawn using a JMI disposable syringe (JMI Syringes and Medical Devices Ltd., Bangladesh) via venipuncture. This blood was then transferred to a non-vacuum blood collection tube (red clot tube) for clotting at room temperature. After 30 min, the whole blood samples were centrifuged at 4400 rpm for 10 min at room temperature using a Sorvall™ ST 8R centrifuge from Thermo Fisher Scientific (Waltham, MA, United States) to separate the clots. The resulting serum samples were collected and stored in a laboratory refrigerator at −20°C for subsequent analysis.

FBS, ALT, serum creatinine, and lipid profile (total cholesterol, triglycerides, high-density lipoprotein cholesterol, and low-density lipoprotein cholesterol) were all assessed using the HumaLyzer 3,000 semi-automated biochemistry analyzer (Wiesbaden, Germany). These tests were conducted using commercially available serum colorimetric methods and kits from Human Diagnostics Worldwide (Wiesbaden, Germany), following the manufacturer’s instructions.

### Diagnostic criteria

We measured serum fasting blood glucose (FBS) levels to identify whether participants were hyperglycemic or had normal blood glucose levels. An FBS level below 100 mg/dL (<5.6 mmol/L) is considered normal, while hyperglycemia is defined as an FBS level above 125 mg/dL (>6.9 mmol/L) (36). Abnormal or elevated liver enzyme was confirmed if the ALT level > 45 U/L in men and > 34 U/L in women (37). For adult males, the normal range for serum creatinine is between 0.78 and 1.25 mg/dL (69–111 micromoles/L), while for adult women, the normal range is between 0.64 and 1.07 mg/dL (57–95 micromoles/L) (38). The National Cholesterol Education Program Adult Treatment Panel III (NCEP/ATP III) was followed to define hyperlipidemia, as the presence of one or more measurements of total cholesterol (TC) ≥ 200 mg/dL; triglyceride (TG): ≥ 150 mg/dL; low-density lipoprotein cholesterol (LDL-C) ≥ 130 mg/dL and high-density lipoprotein cholesterol (HDL-C) < 40 mg/dL (39). Body mass index (BMI) was categorized as normal weight (18.5–22.9 kg/m^2^), overweight (23–24.9 kg/m^2^), and obese (≥25 kg/m^2^) (40, 41).

### Statistical analysis

Statistical analysis was conducted using SPSS version 22.0 (IBM, Chicago, IL, United States). Exploratory data analysis was employed to summarize and present the baseline characteristics of the data. Categorical variables were expressed as percentages (%), while continuous variables were presented as mean ± SD. An independent sample *t*-test was used to determine differences between two groups for continuous variables, and a chi-square test was applied to categorical data. A paired *t*-test was utilized to assess the significance of data collected from the same individuals under two different conditions. Shapiro–Wilk Test and Q-Q plot were used to check if the data were normally distributed. Pearson correlation was used to assess the linear relationship between two continuous variables. Fisher’s exact test was used for absolute frequencies lower than 5 to evaluate differences between groups. *p* ≤ 0.05 indicates statistical significance in our analysis.

## Results

### Characteristics of study participants

In this study, a total of 133 participants were enrolled, with 44 (33.08%) reported being infected and 89(66.92%) non-infected by SARS-CoV-2. The quantitative variables for both groups are presented in Table 1. The mean levels of TC (178.51 ± 47.50) and LDL-C (123.48 ± 43.37) were higher in the infected participants, while the mean levels of ALT (36.70 ± 16.13) and TG (166.53 ± 74.36) were higher in the non-infected participants. However, these differences were not statistically significant. Additionally, the mean levels of FBS, age, BMI, creatinine, and HDL-C were nearly identical between the two groups (Table 1).

**Table 1:** Distribution of the quantitative variables studied during COVID-19 pandemic based on self-reported SARS-CoV-2 infection.

Baseline parameters	Infected n (%)	Non-infected n (%)	Total n (%)	*p* value
Number of participants	44 (33.08)	89 (66.92)	133 (100)	–
Age	44.57 ± 6.83	44.66 ± 5.53	44.63 ± 5.98	0.932
BMI (kg/m2)	25.78 ± 2.35	25.45 ± 2.60	25.56 ± 2.52	0.478
FBS (mmol/l)	5.83 ± 1.66	5.70 ± 1.39	5.75 ± 1.48	0.625
ALT (U/L)	35.66 ± 12.80	36.70 ± 16.13	36.36 ± 15.07	0.710
Creatinine (mg/dl)	0.966 ± 0.18	0.973 ± 0.14	0.97 ± 0.15	0.809
TC (mg/dl)	178.51 ± 47.50	173.85 ± 38.89	175.39 ± 41.81	0.547
TG (mg/dl)	163.97 ± 88.40	166.53 ± 74.36	165.69 ± 78.95	0.861
HDL-C (mg/dl)	33.63 ± 8.85	33.66 ± 8.99	33.65 ± 8.91	0.986
LDL-C (mg/dl)	123.48 ± 43.37	114.41 ± 31.65	117.42 ± 36.04	0.173

Data are presented as mean ± SD or %. *p*-values are obtained from independent sample t-test for continuous variables.

Table 2 shows the frequencies of the qualitative variables for both the infected and non-infected participants. The percentage of having CVD (11%, *p* = 0.36), overweight (38.64%, *p* = 0.07), elevated creatinine level (4.55%, *p* = 0.46), elevated TC level (36.36%, *p* = 0.21), elevated LDL-C level (43%, *p* = 0.08), elevated HDL-C level (63.64%, *p* = 0.43) was higher in infected participants compared to non-infected self-repot. Elevated FBS and TG level frequency were almost the same for infected and non-infected participants.

**Table 2:** Frequency distribution of the studied qualitative variables during the COVID-19 pandemic based on self-reported SARS-CoV-2 infection.

Baseline parameters	Infected n (%)	Non-infected n (%)	Total n (%)	*p* value
Number of participants	44 (33.08)	89 (66.92)	133 (100.00)	–
Sex				0.825
Male	39 (88.60)	80 (89.90)	119 (89.47)	
Female	5 (11.40)	9 (10.10)	14 (10.53)	
Age (years)				
30–35	5(11)	3 (3)	8 (6.01)	0.273
36–40	5(11)	15(17)	20(15.03)	
41–45	14(32)	36(40)	50(37.59)	
46–50	10(23)	22(25)	32(24.06)	
51–55	8(18)	12(14)	20(15.03)	
56–60	2(5)	1(1)	3(2.25)	
Health precautions				
Strictly maintained	16 (36.36)	28 (31.46)	44(33.08)	0.572
Roughly maintained	28 (63.63)	61(68.54)	89(66.92)	
BMI(Kg/m^2^)				
Normal	3(6.82)	17(19.19)	20(15.04)	0.072
Overweight	17(38.64)	21(23.60)	38 (28.57)	
Obese	21(47.73)	51(57.30)	42 (31.58)	
Vaccination status				
No	–	–		
Yes	44(33.08)	89 (66.92)	100	
CVD				
No	39 (88.6)	83 (93.3)	122 (91.73)	0.363
Yes	5 (11.4)	6 (6.7)	11(8.27)	
Elevated creatinine level				
Yes	2(4.55)	2(2.24)	4(3)	0.599
No	42 (95.45)	87(97.75)	129(96.99)	
Elevated FBS level				
Yes	11(25)	23(25.84)	34(25.56)	0.917
No	33(75)	66(74.16)	99(74.44)	
Elevated TG level				
Yes	20(45.45)	44(44.94)	60(45.11)	0.665
No	24(54.54)	45(55.06)	73(54.89)	
Elevated TC level				
Yes	16(36.36)	23(25.84)	39(29.32)	0.210
No	28(63.64)	66(74.16)	94(70.68)	
Elevated LDL-C level				
Yes	19(43.18)	25(28.09)	44(33.08)	0.082
No	25 (56.82)	64(71. 91)	89(66.92)	
Low HDL-C level				
Yes	33(63.64)	72(59.55)	81(60.90)	0.432
No	11(36.36)	17(40.45)	52(39.09)	

Data are presented as mean ± SD or %. *p*-values are obtained from the chi-square test for categorical variables.

### Health precautions, self-reported SARS-CoV-2 infection, and mean BMI distribution across different age groups

**Figure 1:**
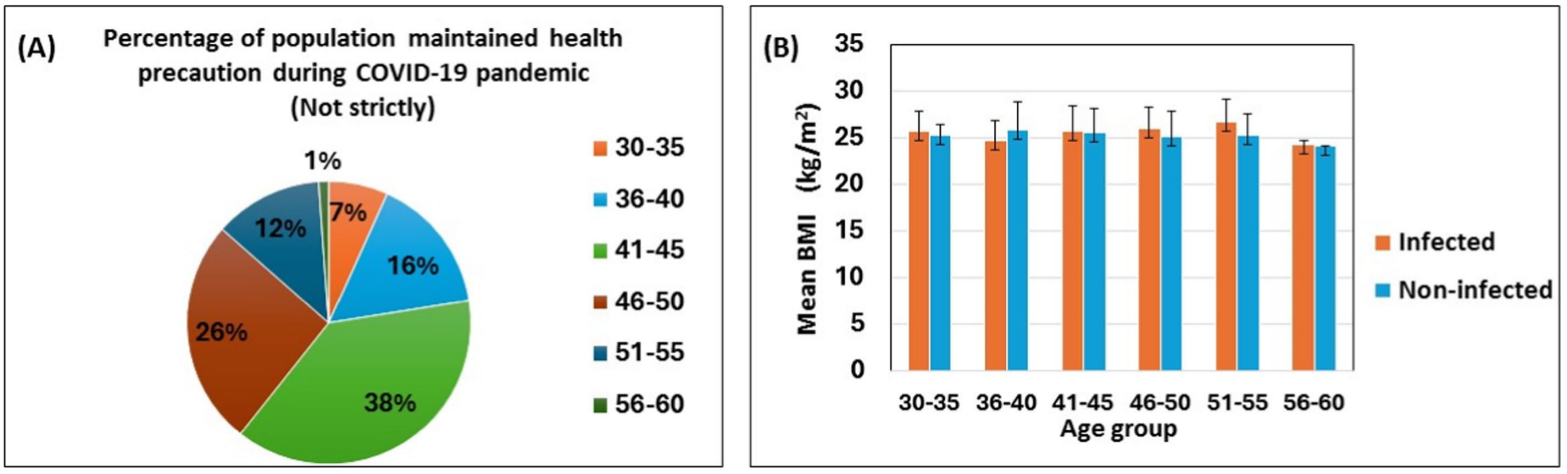
Self-reported SARS-CoV-2 infection with health precaution, age and body mass index. The pie chart shows the percentages of the participants following the health precautions during the COVID-19 pandemic **(A)**. The bar diagram shows the association between age groups, mean body mass index, and infected vs. noninfected populations **(B)**. Data are represented as mean ± standard deviation and percent (%).

Participants were categorized into six age groups, and the percentage of COVID-19 infections by age group is shown in Table 2 and Figure 1. The SARS-CoV-2 infection was higher in the middle-aged group from 41 to 45 (32%). The 46–50 age group had the second highest percentage (23%) of affected participants. The SARS-CoV-2 infection was found to be less common in adults over the age of 56 (5%), according to our findings (Table 2). People between the ages of 41 to 45 did not strictly follow (38%) the necessary safety measures (Figure 1A) during the pandemic period. In addition, only 1% of people aged 56–60 maintained health precaution roughly. This might clarify why this group has the lowest percentage of COVID-19 infections.

Figure 1B depicts the mean BMI of self-reported SARS-CoV-2 infected and non-infected participants across different age groups. The mean BMI is similar for both groups; however, infected participants, except for those in the 36–40 age group, tend to have a slightly higher mean BMI compared to their non-infected counterparts (Figure 1B).

### Different biomarker levels in SARS-CoV-2 infected participants

In this study, different biomarker levels were estimated among the participants before and after COVID-19 pandemic. Out of 133 participants, 44 reported having been infected. The data of infected individuals with increased biomarker levels following COVID-19 pandemic are shown in Table 3 as mean ± standard deviation with *p* value. Interestingly, it was seen that, at the post-pandemic stage, the mean levels of FBS (6.18 ± 1.88), creatinine (1.08 ± 0.14), ALT (42.50 ± 10.81), TC (197.46 ± 43.65), and LDL-C (142.07 ± 45.51) increased greatly with *p* values of 0.015, <0.001, <0.001, 0.026, and 0.008, respectively. However, following infection, the mean HDL-C value was significantly decreased (29.81 ± 7.19) compared to pre-pandemic HDL-C (37.89 ± 7.34).

**Table 3:** Comparison of biomarker levels before and after the COVID-19 pandemic.

Variables	Pre-pandemic (*n* = 44)	Post-pandemic (*n* = 44)	*p* value
Creatinine(mg/dL)	0.9105 ± 0.12	1.08 ± 0.14	< 0.001
FBS (mmol/L)	5.14 ± 1.28	6.18 ± 1.88	< 0.001
ALT(U/L)	30.63 ± 7.63	42.50 ± 10.81	< 0.001
TC (mg/dL)	166.09 ± 44.32	197.46 ± 43.65	< 0.001
TG (mg/dL)	151.33 ± 79.45	210.711 ± 101.89	0.001
LDL-C(mg/dL)	106.95 ± 35.68	142.07 ± 45.51	< 0.001
HDL-C(mg/dL)	37.89 ± 7.34	29.81 ± 7.19	0.002

The data are represented as mean standard deviation ± (SD). The paired *t*-test is used to get the *p* values for the comparison among the population in pre-pandemic and post-pandemic stage.

### Lipid profile and liver enzyme relationship in SARS-CoV-2 infected population

**Figure 2:**
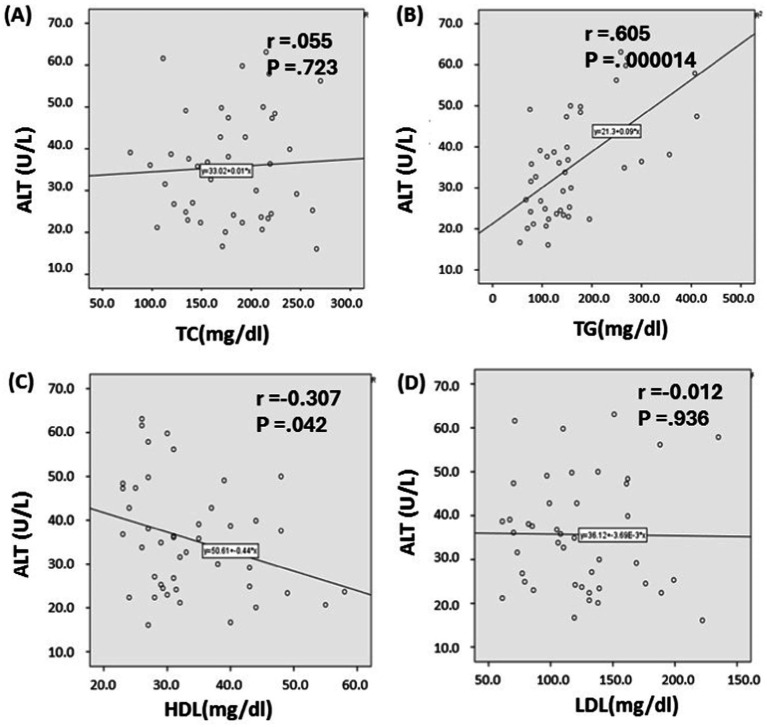
Relationship between lipid profile and serum alanine transaminase Levels in SARS-CoV-2 infected individuals. Based on the scatter plot, it seems that there is a connection between alanine transaminase (ALT) and triglycerides (TG), as well as between ALT and high-density lipoprotein cholesterol (HDL-C) **(B, C)**. Whereas, Total cholesterol (TC) and Low-density lipoprotein cholesterol (LDL-C) shows no association with ALT level **(A, D)**. *p* ≤ 0.05 indicates significance.

The scatterplot (Figure 2) describes the correlation that exists between the lipid profile and the liver enzyme alanine transaminase (ALT) in a population that self-reported SARS-CoV-2 infection. It was observed that there is a significant and positive correlation between ALT and TG (*r* = 0.605, *p* < 0.001; Figure 2B). ALT and HDL-C were discovered to have an inverse correlation with each other (*r* = 0.307, *p* = 0.042; Figure 2C).

### Lipid profile and liver enzyme relationship in SARS-CoV-2 non-infected population

Supplementary Figure S1 illustrates the scatterplot of the lipid profile and ALT level in individuals who did not report SARS-CoV-2 infection. The *p* values for ALT vs. TC, TG, LDL, and HDL are 0.989, 0.643, 0.689, and 0.470, respectively. These findings indicate that there was no statistically significant positive correlation observed between ALT and any of the lipid profile parameters (TG, TC, HDL-C, or LDL-C).

### Effect of COVID-19 vaccination on different serum biomarkers

**Figure 3:**
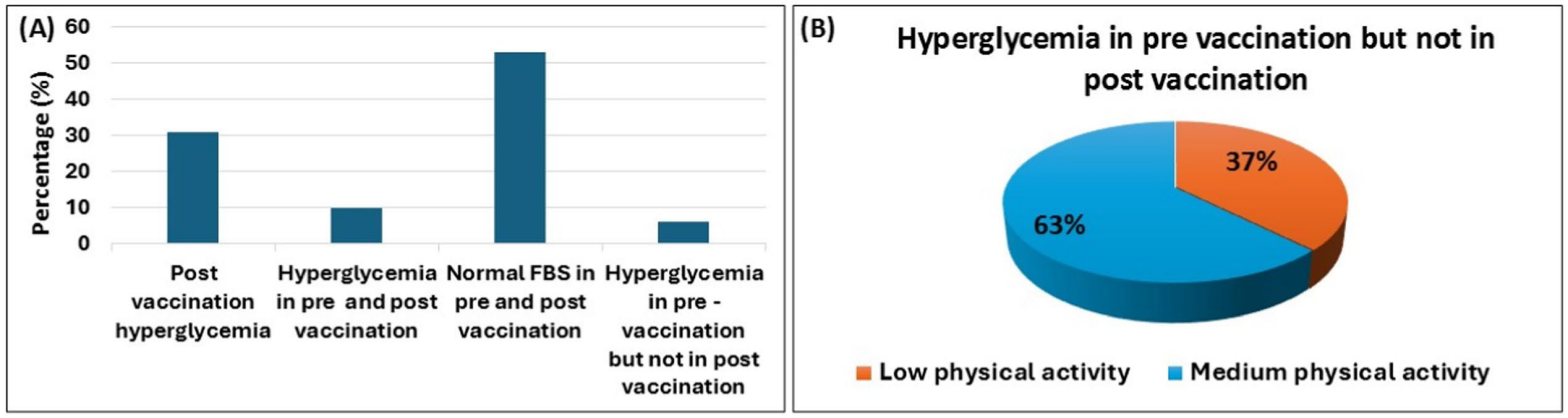
COVID-19 vaccination and hyperglycemia. The bar diagram illustrates the changes in hyperglycemia prevalence among participants during the pre-vaccination and post-vaccination periods **(A)**. It also highlights physical activity levels (from low to medium) in hyperglycemic individuals who transitioned to a non-hyperglycemic state after vaccination **(B)**. Data are represented as percent (%).

In Bangladesh, mRNA, viral vector, and inactivated virus vaccines were administered. All participants in this study received different types of COVID-19 vaccines. To assess the impact of vaccination on various serum biomarkers, we did not differentiate between the types of vaccines administered. Vaccination data was collected from participants at the time of blood sample collection, both before the vaccination started and after the two doses of vaccination. Hyperglycemia was diagnosed based on FBS levels that were measured before and after the COVID-19 vaccination. Figure 3A shows that, following COVID-19 vaccination 31% of participants who were non-hyperglycemic prior to vaccination, were newly diagnosed with hyperglycemia. Conversely, 6% of the population had hyperglycemia before vaccination but not afterward. Considering data on physical activity levels, participants who remained physically active (at a moderate level) were more likely to maintain normal blood glucose levels after vaccination than those who had hyperglycemia prior to vaccination, as shown in Figure 3B.

Table 4 represent the biomarker level of pre and post vaccination with mean ± SD. The paired *t*-test indicated a significant increase in serum creatinine (0.971 ± 0.16) and FBS (5.75 ± 1.45) levels following vaccination (*p* ≤ 0.05). However, the average TC level (175.35 ± 41.81) reduced considerably following vaccination (*p* value is in border line). The mean ALT (38.14 ± 21.29 vs. 36.37 ± 15.08) and HDL-C (32.73 ± 9.34 vs. 33.64 ± 8.913) levels were nearly same in both scenarios (pre vs. post vaccination).

**Table 4:** Comparison of biomarker levels in pre and post vaccination during the COVID-19 pandemic.

Variables	Pre-vaccination (*n* = 133)	Post-vaccination (*n* = 133)	*p* value
Creatinine(mg/dL)	0.93 ± 0.13	0.971 ± 0.16	0.022
FBS (mmol/L)	5.28 ± 1.2	5.75 ± 1.45	0.006
ALT(U/L)	38.14 ± 21.29	36.37 ± 15.08	0.441
TC (mg/dL)	184.02 ± 41.48	175.35 ± 41.81	0.050
TG (mg/dL)	171.89 ± 79.80	165.69 ± 78.96	0.542
LDL-C(mg/dL)	123.67 ± 34.44	117.42 ± 117.42	0.128
HDL-C(mg/dL)	32.73 ± 9.34	33.64 ± 8.913	0.420

The data are represented as mean standard deviation ± (SD). The paired *t*-test is used to get the *p* values for the comparison among the population in pre-vaccination and post-vaccination stage.

## Discussion

This study compared changes in various serum biomarkers before and after the COVID-19 pandemic and vaccination among academic and non-academic participants at Shahjalal University of Science and Technology, Sylhet, Bangladesh.

In our study, SARS-CoV-2 infected group were determined by their responses during data collection, as they have different COVID-19 like symptoms and diagnosed positive by RT-PCR as well. In contrast, the non-infected group were clinically diagnosed negative by RT-PCR, undiagnosed, and may or may not be infected with SARS-CoV-2, as they have no symptoms. Therefore, Table 1 shown no substantial variation of serum biomarker levels between the two groups.

While individuals of all ages can be affected by COVID-19, older people (>65 years) are more vulnerable to severe illness due to physiological changes and health issues related to age. Recent data indicates an increased susceptibility to infection, and mortality rates elevate significantly with age, with minimal deaths occurring before age 50 (42–44). Our study implies that adults aged 41 to 45 have a higher prevalence of SARS-CoV-2 self-reported infection, potentially impacted by their physical activity, lifestyle, and adherence to health precautions during the pandemic.

Lifestyle and food habit are related to the BMI of the participants and according to 2018 WHO data, 13 percent of adults (>18 years) were obese worldwide in 2016 (45). Different study reported that people with overweight or obese had greater rates of COVID-19 than people who were healthy BMI (46). This link remained consistent regardless of age, gender, or other comorbidities like high blood pressure, diabetes, or dyslipidemia. In this study, we reported that participants with self-reported SARS-CoV-2 infection had a higher percentage of overweight individuals (38.64%) than without the infection (23.60%). In our study, though both infected and non-infected participants had a BMI exceeding 25 kg/m^2^, infected people had a slightly higher BMI, as the overweight individuals are more susceptible to acute and chronic diseases and become more sensitive to COVID-19 (47). Obesity seems to impact the major immune cell types involved in responding to SARS-CoV-2. When a person’s BMI increases, anti-inflammatory CD4+ T-cell subsets like Th2 and T regulatory cells become more active. Since controlling viral spread relies on inflammatory responses, increased anti-inflammatory cell activity could hinder this process (48). Previously it was reported that MERS-CoV infection was more likely in patients with underlying cardiovascular disease (CVD) (49). CVD was also found to be linked with the three-fold increased risk of severe SARS-CoV-2 infection (50), and it supports our finding that the CVD individuals are more prone to SARS-CoV-2 infection.

A retrospective investigation revealed that people with diabetes had a considerably elevated fatality rate and a three-fold increased risk of death than those without diabetes (51). Because of the reduction of T cell response, function of neutrophil, and humoral immune response, a hyperglycemic environment raises vulnerability to most of the types of infection (52). In our study, FBS level significantly increased in the post pandemic stage (*p* < 0.05). These elevations could lead to long-term hyperglycemia, making these individuals more susceptible to developing diabetes.

Some individuals affected by the 2009 H1N1 virus showed modest to moderate increases of blood creatine kinase (53), and the prevalence of the disease varies by person. However, a recent study from a Wuhan high complexity reference hospital looked at 702 COVID-19 patients and discovered that 18% had elevated serum creatinine levels (54). Our study showed that participants with SARS-CoV-2 infection had a statistically significant increase in their serum creatinine levels. Elevated creatinine level insinuates that renal damage may occur due to SARS-CoV-2 infection.

The high levels of angiotensin-converting enzyme 2 (ACE) can be explained by the high concentrations of the ACE2 receptor that are in liver and bile duct cells. SARS-CoV-2 may directly connect to ACE2 present on cholangiocytes causing liver damage (55). Several studies have indicated that liver injury can occur in patients with SARS, primarily characterized by mild to moderate elevations in alanine aminotransferase (ALT) levels (56, 57). COVID-19 patients (*N* = 1,099) were spliced into groups based upon their AST and ALT levels and majority of them occurred in severe and catastrophic cases, with 39.4% having AST level greater than 40 U/L and 28.1% having ALT level greater than 40 U/L (58). According to our findings, ALT level went up significantly after SARS-CoV-2 infection in infected population, which infers that gradual liver destruction is associated with SARS-CoV-2 infection.

Lipids are essential cellular components in the lifecycle of SARS-CoV-2, playing key roles in processes such as endocytosis, exocytosis, viral replication, and fusion with host cells (59). Lipid rafts and cholesterol are particularly important during the initial stages of infection (60). Recent studies have shown that SARS-CoV-2 infection leads to decreased levels of HDL-C and increased levels of triglycerides (TG) (61). Another study found that the infection significantly altered all lipid markers, with common changes including elevated TG levels and reduced total cholesterol (TC) (62, 63). Additionally, follow-up research on COVID-19 patients reported a significant increase in LDL-C levels (64). Our findings are consistent with these observations, showing that individuals who self-reported SARS-CoV-2 infection and had elevated biomarker levels experienced significant changes in all lipid profile parameters in the post-pandemic era.

Further, in this study we observed the correlation between liver enzyme and lipid profile in both SARS-CoV-2 infected and non-infected people. A study revealed that the correlation between liver enzymes and lipid markers in persons with type 2 diabetes was investigated and found that ALT has a substantial positive association with TG, TC, and LDL-C, but a negative correlation with HDL-C (65). Whereas we observed that ALT has a substantial relationship with TG and a mild association with HDL-C among the SARS-CoV-2 infected populations.

Multiple COVID-19 vaccines have already been licensed for urgent use across the world. Although the vaccinations’ efficacy is undeniable, their safety remains a concern. The mRNA vaccine had been under development and refinement for about two decades, but it was only recently deployed in clinical trials (66). Another reported that, only 0.6 percent of participants in the BNT162b2 mRNA study had liver damage (67). Moreover, one case study showed that an individual with an ALT level of 707 U/L had acute liver damage after receiving mRNA-1273 vaccination (68). In this study, we assessed the impact of vaccination on various serum biomarkers without differentiating between the types of vaccines administered. However, we found that the mean ALT levels remained nearly identical in study participants before and after vaccination.

After receiving the BNT162b2 and mRNA-1273 vaccines, new incidence of glomerulonephritis (GN) was discovered. Immunoglobulin A Nephropathy (IgAN) has been the most reported GN so far (69). However, the IgA antibody (Ab) production and deposition inside the kidneys are not clearly associated with COVID-19 vaccination. In this study, the mean serum creatinine level elevated significantly following the administration of different COVID-19 vaccines.

Patient having obesity and dyslipidemia experience hyperglycemia after receiving a first dose of the Covishield (AstraZeneca) vaccine (70) and vaccination-induced hyperglycemia (ViHG) was also found after taking BNT162b2 and mRNA-1273 vaccine (71, 72). The majority of the participants’ FBS levels were normal before and after vaccination, according to our findings. After receiving the COVID-19 vaccination, only 31% of the population had hyperglycemia and this could be due to the fact that these individuals had pancreatic damage or acute pancreatitis prior to vaccination. Immune response will begin after vaccination and the body will require additional energy and some excess glucose may be produced, causing blood sugar levels to rise. The FBS level also rose significantly after vaccine administration compared to the pre-vaccination stage.

A study assessing the biochemical effects of COVID-19 vaccinations found no significant changes in lipid profile levels (73). However, our research revealed a significant reduction in the mean level of total cholesterol (TC) after vaccination, while the mean level of HDL-C remained relatively unchanged compared to pre-vaccination levels. In our study, many participants reported being infected with SARS-CoV-2, and several had chronic conditions that were managed with medication. These factors may influence the outcomes of post-vaccination results.

Despite the relatively small sample size of this cohort study, it provides valuable insights into the impact of SARS-CoV-2 infection and vaccination on several early biomarkers among participants in Sylhet, Bangladesh. However, there are some limitations to our study. The small sample size hindered the accurate calculation of probabilities and risk factors. Various confounding variables may have influenced the observed correlations. Additionally, self-reported data on SARS-CoV-2 infection could be impacted by memory recall bias, affecting data accuracy. The study primarily included men and was conducted in a specific suburban area, highlighting the need for research involving a larger and more diverse population. Furthermore, there may have been unidentified risk factors that significantly affected the results. To better understand the changes in biomarker levels during COVID-19 pandemic and vaccination, further research is necessary. We plan to use multilevel modeling in future studies to achieve more robust outcomes.

## Conclusion

In conclusion, our study found that serum biomarker levels can increase following COVID-19 pandemic, with ALT levels showing a significant positive correlation with TG levels in self-reported SARS-CoV-2 affected individuals. Vaccination appears to influence FBS, creatinine, and TC levels. Therefore, routine monitoring of liver function, kidney function, blood sugar, and lipid profiles is crucial for the early detection of liver impairment, renal and pancreatic dysfunction, and cardiovascular events in individuals who have been infected with or vaccinated against SARS-CoV-2, to better understand their long-term pathophysiological effects. Further research is also needed to investigate other clinical markers to deepen our understanding of the long-term consequences of SARS-CoV-2 infection and vaccination.

## Data Availability

The raw data supporting the conclusions of this article will be made available by the authors, without undue reservation.

## Glossary

SARS-CoV-2: Severe acute respiratory syndrome coronavirus 2
COVID-19: Coronavirus disease
ARDS: Acute respiratory stress syndrome
SARS-CoV-1: Severe Acute Respiratory Syndrome coronavirus-1
MERS-CoV: Middle East Respiratory Syndrome coronavirus
RT-PCR: Reverse transcription-polymerase chain reaction
AKI: Acute kidney injury
BUN: Blood urea nitrogen
ACE-2: Angiotensin converting enzyme 2
TMPRSS2: Transmembrane protease, serine 2
MOF: Multiple organ failure
FBS: Fasting blood sugar
TG: Triglyceride
TC: Total cholesterol
HDL-C: High density lipoprotein cholesterol
LDL-C: Low density lipoprotein cholesterol
ALT: Alanine aminotransferase
BMI: Body mass index
CVD: Cardiovascular disease
WHO: World health organization
GN: Glomerulonephritis
IgAN: Immunoglobulin A Nephropathy
ViHG: Vaccination-induced hyperglycemia
