# Supplementary material for: Assessment of serum biomarker changes following the COVID-19 pandemic and vaccination: a cohort study in Sylhet, Bangladesh
Source: Front Public Health. 2025 Feb 21;13:1435930. doi: 10.3389/fpubh.2025.1435930 (PMC11885237; doi:10.3389/fpubh.2025.1435930)
Supplement: Supplementary file 1 [file Data_Sheet_1.docx]

**Frontiers in Public Health**

**Title: Assessment of serum biomarker changes following the COVID-19 pandemic and vaccination: a cohort study in Sylhet, Bangladesh**


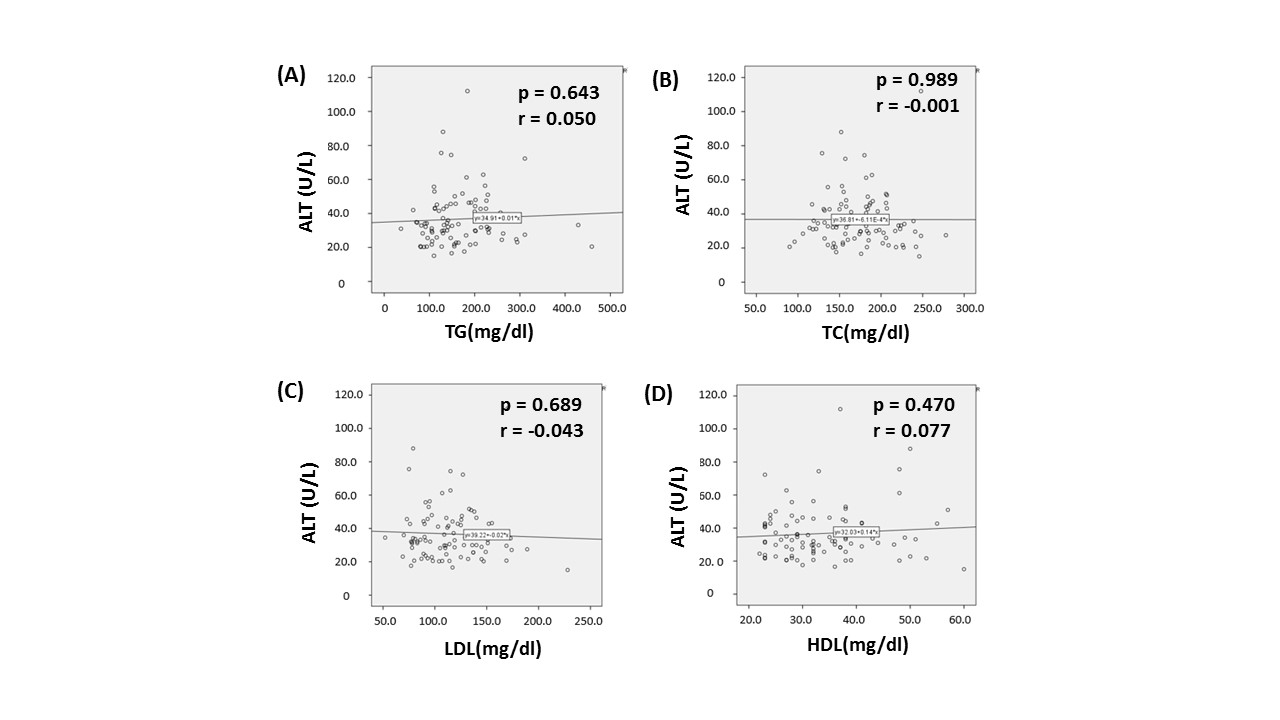


**Figure S1: Relationship between lipid profile and serum Alanine transaminase Levels in COVID-19 non-infected individuals.** The scatter plot illustrates that all the *P* values are greater than 0.05, indicating that the lipid profile and Alanine transaminase have no statistically significant relationship.
